# Selection of Comprehensive Assessment Categories Based on the International Classification of Functioning, Disability, and Health for Elderly Patients with Heart Failure: A Delphi Survey among Registered Instructors of Cardiac Rehabilitation

**DOI:** 10.1155/2021/6666203

**Published:** 2021-06-25

**Authors:** Shigehito Shiota, Makiko Naka, Toshiro Kitagawa, Takayuki Hidaka, Naoki Mio, Kana Kanai, Mariko Mochizuki, Hiroaki Kimura, Yasuki Kihara

**Affiliations:** ^1^Heart Failure Center, Hiroshima University Hospital, Hiroshima, Japan; ^2^Department of Rehabilitation, Division of Clinical Support, Hiroshima University Hospital, Hiroshima, Japan; ^3^Department of Cardiovascular Medicine, Hiroshima University Graduate School of Biomedical and Health Sciences, Hiroshima, Japan; ^4^Hiroshima Integrated Community Care System Promotion Center, Hiroshima, Japan; ^5^Department of Rehabilitation Medicine, Hiroshima University Hospital, Hiroshima, Japan; ^6^Hiroshima University Graduate School of Biomedical and Health Sciences, Hiroshima, Japan

## Abstract

The development of a comprehensive assessment tool based on the International Classification of Functioning, Disability, and Health (ICF) for elderly patients with heart failure is urgently required. In this study, we classified the ICF categories relevant to heart failure in the elderly through a Delphi survey (3-step questionnaire survey) of 108 Registered Instructors of Cardiac Rehabilitation in the Hiroshima Prefecture. Questionnaires were conducted using postal mail or a web-based platform. The survey was conducted three times, and the survey results were provided as feedback to the participants in the second and third rounds. More than 80% of the respondents selected categories according to the ICF core set methodology. Data were collected from December 2018 to March 2019, with 67, 54, and 46 participants in the first, second, and third rounds, respectively. A total of 58 ICF items were adopted based on the results: 27 body function items, 4 body structure items, 20 activity and participation items, and 7 environmental factor items. This study is characterised by the inclusion of a large number of ICF items for mental function. This result seems to be influenced by the increasing interest in cognitive dysfunction in elderly patients with heart failure. The ICF categories selected for this study allow for a comprehensive assessment of clients for occupational therapy. The findings of this study are expected to provide a basis for an outcome measure to determine the effectiveness of occupational therapy for these patients.

## 1. Introduction

The elderly population with heart failure is increasing globally with the increase in the aging population, and the number of patients with heart failure is estimated to exceed 1.3 million by 2030 in Japan [1]. Previous studies have shown that approximately 35% of patients with heart failure are rehospitalised within 1 year [2]. Many lifestyle-related factors, such as high salt and water intake, poor medication adherence, overwork, and physical and mental stress, are known to exacerbate heart failure [3]. Furthermore, comprehensive factors that are reported to be associated with readmission include physical functioning, such as exercise tolerance [4] and walking speed [5]; mental functioning, such as cognitive functioning [6], depression [7], and anxiety [8]; personality [9], activity and participation, such as activities of daily living [10], instrumental activities of daily living (IADL) [5], and leisure [11]; and social support [12]. Comprehensive cardiac rehabilitation that is managed by a multidisciplinary team reduces mortality and readmissions in elderly patients with heart failure [13–19]. In Japan, there is a qualifying program for the provision of comprehensive cardiac rehabilitation: the Registered Instructors of Cardiac Rehabilitation (RICRs), issued by the Japanese Association of Cardiac Rehabilitation (JACR). The RICRs are accredited by the JACR and consist of doctors, nurses, physiotherapists, occupational therapists, management dietitians, pharmacists, and clinical psychologists. As part of the multidisciplinary team, occupational therapists have the responsibility to promote the quality of life and health of elderly patients with heart failure. In multidisciplinary team management, it is important to share not only disease information but also information on functioning, disability, environmental factors, and personal factors based on the International Classification of Functioning, Disability, and Health (ICF) framework [20]. The ICF is a health and health-related framework, published by the World Health Organization (WHO) in 2001, which classifies human life into approximately 1500 codes. The ICF is strongly relevant from the perspective of occupational therapy. In Japan, the Japanese Association of Occupational Therapists has developed the Management Tool for Daily Life Performance (MTDLP) by utilising the ICF framework and has reported its effect on quality-adjusted life-year improvement for elderly participants in a randomised controlled trial (RCT) [21–23]. In an RCT of patients with cardiovascular disease, Fukui et al. showed that the MTDLP improves depression and IADL [24].

The ICF is an effective tool for the management of occupational therapy for elderly patients with heart failure; however, the ICF has not been used sufficiently in clinical practice in Japan. To address the complexity of the ICF codes, the WHO developed the ICF core set [25], which is a short list of evidence-based ICF categories that reflect the spectrum of typical problems that are experienced by patients with a particular health condition. The ICF core set is a collection of information based on the literature, expert opinion, and empirical information that will be further developed by using information from research and patient qualitative studies. Thus far, several ICF core sets have been developed [26–28]; however, there is no ICF core set that is specific to heart failure in elderly patients. In addition, due to the differences in medical systems in each country, evaluation tools developed in other countries may not be suitable for the Japanese medical and social systems. Therefore, it is necessary to develop assessment tools by using the ICF adapted to the Japanese culture and medical system.

This study was conducted with the aim to collect expert (RICR) opinions to select ICF categories for the comprehensive assessment of elderly patients with heart failure.

## 2. Materials and Methods

### 2.1. Study Design

**Figure 1:**
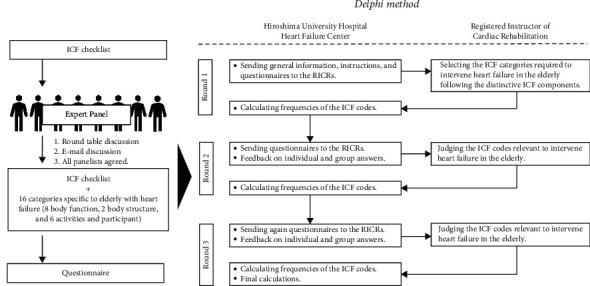
Schematic representation of the research process.

We conducted a prospective questionnaire survey by using the Delphi method to select ICF categories for the comprehensive assessment of elderly patients with heart failure. Three rounds of mail and web-based questionnaires were administered to RICRs in accordance with the Delphi method, which is a structured consensus-building method with four characteristics: anonymity, repetition with controlled feedback, statistical group responses, and input from experts [29–31].  Figure 1 shows a flowchart describing the research process.

### 2.2. Participants

The study participants were 108 RICRs from the Hiroshima Prefecture. The participants were listed with their names and affiliations on the JACR website and were registered by the Heart Failure Center (HFC).

### 2.3. Data Collection and Measures

#### 2.3.1. Preparation of the Questionnaire

The ICF checklist is a tool designed for the clinical use of the ICF and consists of a face sheet, listening item assessment, health information, general questions about activity and participation, and guidelines for use. We prepared a questionnaire based on the ICF checklist by following the methodology of the ICF core set [28]. An expert panel comprising multidisciplinary heart failure team members (two cardiologists: T.K. and T.H.; two nurses: M.N. and H.T.; two physiotherapists: K.K. and N.M.; one occupational therapist: S.S.; and one care manager: M.M.) discussed the ICF categories specific to heart failure that were to be added to the ICF checklist. The expert panel members had sufficient experience in medical care and rehabilitation of elderly patients with heart failure. The expert panel conducted round-table and e-mail discussions until they all agreed on the survey items.

#### 2.3.2. Delphi Survey among RICRs

In the first round, we sent general information, instructions, and questionnaires to 108 RICRs. The RICRs responded to the HFC with a selection of ICF categories deemed necessary for intervention in elderly patients with heart failure. The HFC calculated the selection rate of ICF categories.

In the second round, we sent again the same questionnaire as that in the first round to the participants along with the results of the first-round survey. The participants selected the ICF categories relevant to elderly patients with heart failure, and they responded through Google Forms or a reply envelope. The HFC calculated the selection rate of ICF categories.

In the third round, the HFC sent the same questionnaire again along with the results of the second-round survey. Based on the results of the second round, the RICRs selected the ICF categories required for intervention in elderly patients with heart failure. Thus, in this study, three surveys were conducted using the same questionnaire.

### 2.4. Statistical Analysis

We computed the percentage score after a simple tabulation of the collected data. In reference to a previous report [32], we adopted an ICF item as an assessment item if at least 80% of the respondents answered that it was “necessary.”

### 2.5. Ethical Considerations

This research was approved by the Hiroshima University Epidemiological Research Ethics Review Board (approval number: E-1176). Moreover, we explained to the participants that there were no disadvantages if they did not take part in the surveys, how we would handle their personal information on the survey sheets, and that responding to the survey implied consent for participation in the study.

## 3. Results

### 3.1. Preparation of the Questionnaire

The expert panel prepared the questionnaire and added ICF categories specific to heart failure to the ICF checklist. A total of 143 categories were included in the questionnaire—namely, 39 body function categories (categories specific to heart failure: b126, b172, b250, b45, b455, b460, b545, and b740), 18 body structure categories (categories specific to heart failure: s140 and s150), 54 activity and participation categories (categories specific to heart failure: d155, d177, d230, d240, d420, and d855), and 32 environmental factor categories.

### 3.2. Delphi Survey among RICRs

Data were collected from December 2018 to March 2019. There were 67, 54, and 46 respondents in the first, second, and third rounds, respectively.  Table 1 summarizes the characteristics of participants who responded in all Delphi survey rounds. With respect to profession, 56.5% of the participants were physiotherapists, 28.3% were doctors, and 10.9% were occupational therapists. Acute care wards accounted for 71.7% of facilities, followed by the rehabilitation wards and clinics.

Table 2 shows the consensus process for the ICF categories that were selected by more than 80% of participants from the first to the third Delphi rounds. There were 51, 45, and 58 ICF categories with a consensus of ≥80% per component in the first, second, and third rounds, respectively. Therefore, we adopted 27 body function items, 4 body structure items, 20 activity and participation items, and 7 environmental factor items. Tables 3 – 6 present the results for the ICF categories (body function, body structure, activity and participation, and environmental factors) that were selected by ≥50% of participants in the third round; items with ≥80% consensus are shown in bold.

## 4. Discussion

In this study, through a survey of RICRs, we selected 58 ICF categories for a comprehensive assessment of elderly patients with heart failure. The 58 ICF categories consisted of 27 body function items, 4 body structure items, 20 activity and participation items, and 7 environmental factor items.

### 4.1. Validity of the Research Method

First, we examined the appropriateness of the sample size in this study. The response rate for all rounds of this study was 42.6%, which was higher than that of previous studies where the average response rate for the ICF core set was 20.3 (6–73%) [25]. Second, we examined the validity of the study population. The study participants were RICRs living in a limited area within the Hiroshima Prefecture. Therefore, they had similar knowledge and attitudes, which possibly contributed to their ability to build consensus smoothly during the study. However, limiting the geographical area of the study participants would undeniably have fostered a bias in the responses. Furthermore, there was a bias with regard to the occupation and affiliation of the respondents; therefore, the results need to be interpreted with caution.

### 4.2. Characteristics of ICF Categories Extracted in This Study

The ICF core set for cardiovascular disease includes a core set for chronic ischemic heart disease (CIHD) in patients receiving long-term care [27]. The ICF core set for CIHD consisted of 61 ICF categories: 14 for body function, 1 for body structure, 17 for activity and participation, and 29 for environmental factors. A comparison of the categories in this study with those in the ICF core set for CIHD shows that the assessment set in this study has more body function categories and fewer environmental factors. In particular, the number of mental function items was nine as compared to the three items of the ICF core set of CIHD. A systematic review reported that approximately 43% of patients with heart failure have cognitive disability [33]. Furthermore, if the cognitive disability of heart failure patients is missed during hospitalisation, the risk of rehospitalisation may increase fivefold [6]. The prognostic factors for heart failure (b152: emotional function (depression and anxiety)) were also selected in this study, although their selection did not exceed 80% (78%) of consensus. These results indicate that RICRs consider mental function to be important in clinical practice.

This study is unique in that we selected “d177: decision making” from the activity and participation set. The Japanese Society of Cardiology's 2018 Guidelines for the Management of Acute and Chronic Heart Failure [34] recommend palliative care and advanced care planning (ACP) as a way to facilitate decisions by patients on how to spend the rest of their lives.

Furthermore, the results of this study showed the selection of items “d5: self-care,” “d6: home life,” and “d920: recreation and leisure,” which is similar to the results of the ICF core set for CIHD. Heart failure in elderly patients is often associated with problems such as living alone and caregiving by an elderly caregiver. Previous studies have reported that IADL (e.g., cooking, shopping, and medication management) in heart failure patients is associated with life prognosis and rehospitalisation [35, 36]. In addition, recreation and leisure have been associated with cardiovascular risk and the degree of independence in IADL [11]. Therefore, we think that it was appropriate to choose these ICF codes.

Significantly fewer environmental factors were selected in our study, 9 compared with the 27 items of the ICF core set for CIHD. We infer ICF categories unrelated to elderly patients (e.g., work and friends) because the study questionnaire focused on elderly patients. The ICF items selected by Japanese cardiac rehabilitation experts differed in some categories from the ICF core set developed in other countries. Therefore, we need to develop an evaluation tool that is customised for Japanese cultural and social systems.

### 4.3. Implication for Occupational Therapy Practice

The ICF categories selected for this study allow for a comprehensive assessment of clients for occupational therapy. We can use these ICF categories as a common medium for sharing information among multiple professionals and caregivers. In addition, a comprehensive assessment based on the ICF combined with the MTDLP can provide effective occupational therapy for elderly patients with heart failure. The findings of this study are expected to provide a basis for an outcome measure to determine the effectiveness of occupational therapy for these patients.

### 4.4. Limitations of This Study

This study had some limitations. First, the study participants were RICRs from Hiroshima Prefecture, with physiotherapists accounting for approximately 60% of the study population. Therefore, one should be cautious in generalising the results because there exists the possibility of bias with regard to area and profession. Second, problems with the Delphi method have been identified with respect to the validity of the questionnaire and the risk of inducing consensus. To increase the validity of the questionnaire, we included multidisciplinary teams comprising professionals who are engaged in the provision of comprehensive care for heart failure. In addition, we tried to provide as much fair feedback as possible; however, it is unknown whether the abovementioned factors affected the outcome of the ICF selection. Third, we did not include categories related to personal factors in this study. As personal factors are essential for understanding the health process, they need to be assessed in future studies. Fourth, because we set a cut-off value of 80% consensus for ICF categories in this study, we had to exclude categories with a very similar agreement of 79% or 78%. Fifth, because the purpose of this study was to develop a comprehensive assessment tool for the elderly with heart failure, we did not adhere to the steps prescribed in the development of the ICF core set. These issues suggest the need for careful attention in the development of ICF-based assessment tools. Additionally, further studies are necessary in the future to develop a comprehensive framework for functioning and disability evaluation in elderly patients with heart failure based on the results of this research, to confirm their reliability and effectiveness.

## 5. Conclusions

The ICF categories relevant to elderly patients with heart failure included a total of 58 categories consisting of 27 body function items, 4 body structure items, 20 activity and participation items, and 7 environmental factor items. The development of a functioning and disability evaluation tool that utilises the ICF categories based on this research is necessary. The ICF categories selected for this study allow for a comprehensive assessment of clients for occupational therapy. The findings of this study are expected to provide a basis for an outcome measure to determine the effectiveness of occupational therapy for these patients.

## Figures and Tables

**Table 1:** Characteristics of the participants who answered in all Delphi rounds (*n* = 46).

Characteristics	*n*	%
*Profession*		
Physicians	13	28.3
Physiotherapists	26	56.5
Occupational therapists	5	10.9
Nurses	2	4.3
*Type of facilities*		
Acute care ward	33	71.7
Rehabilitation ward	4	8.7
Clinic	3	6.5
Long-term care ward	1	2.2
Visiting nursing station	1	2.2
Day service center	1	2.2
Others	3	6.5
*Duration of experience of cardiac rehabilitation experts (years)*		
1–5	24	52.2
6–10	18	39.1
≥11	4	8.7

**Table 2:** The consensus process from the first to the third Delphi rounds.

Characteristics	Round 1	Round 2	Round 3
Number of participants (*n*)	108	67	54
Number of respondents (*n*)	67	54	46
Response rate (%)	62.0	80.6	85.2
*ICF categories with a consensus of ≥80% per component*			
Components combined (*n*)	51	45	58
Body function (*n*)	20	19	27
Body structure (*n*)	3	3	4
Activity and participation (*n*)	21	17	20
Environmental factors (*n*)	7	6	7

**Table 3:** International Classification of Functioning, Disability, and Health (ICF) body function categories that were considered relevant by ≥50% of participants in the third round (items in bold with ≥80% consensus).

ICF code	ICF category title	Round 1 (%)	Round 2 (%)	Round 3 (%)
**b410**	**Heart function**	**99**	**96**	**100**
**b455**	**Exercise tolerance function**	**99**	**98**	**100**
**b730**	**Muscle power function**	**99**	**94**	**100**
**b440**	**Respiration function**	**99**	**96**	**98**
**b710**	**Mobility of joint function**	**93**	**86**	**96**
**b114**	**Orientation function**	**88**	**92**	**94**
**b415**	**Blood vessel function**	**90**	**94**	**94**
**b420**	**Blood pressure function**	**97**	**92**	**94**
**b460**	**Sensations associated with cardiovascular and respiratory functions**	**94**	**98**	**94**
**b110**	**Consciousness function**	**81**	**90**	**91**
**b545**	**Water, mineral, and electrolyte balance functions**	**81**	**86**	**91**
**b126**	**Temperament and personality functions**	67	**80**	**89**
**b430**	**Haematological system function**	**84**	**80**	**89**
**b740**	**Muscle endurance function**	**88**	**90**	**89**
**b164**	**Higher-level cognitive functions**	**85**	**90**	**87**
**b530**	**Weight maintenance functions**	**81**	**80**	**87**
**b620**	**Urination function**	**91**	**84**	**87**
**b130**	**Energy and drive function**	**84**	**84**	**85**
**b140**	**Attention function**	73	76	**85**
**b144**	**Memory function**	**81**	78	**85**
**b515**	**Digestive function**	72	71	**83**
**b117**	**Intellectual function**	**81**	**86**	**80**
**b134**	**Sleep function**	70	78	**80**
**b210**	**Sight function**	73	78	**80**
**b235**	**Vestibular function**	70	65	**80**
**b435**	**Immunological system function**	66	63	**80**
**b525**	**Defecation function**	**82**	78	**80**
b152	Emotional function	64	75	78
b280	Sensation of pain	73	75	78
b230	Hearing function	69	71	76
b555	Endocrine gland function	60	61	74
b250	Taste function	48	53	72
b735	Muscle tone function	64	57	67
b167	Mental functions of language	57	63	63
b310	Voice function	46	49	63
b765	Involuntary movement function	54	45	57
b172	Calculation function	42	45	54

**Table 4:** International Classification of Functioning, Disability, and Health (ICF) body structure categories that were considered relevant by ≥50% of participants in the third round (items in bold with ≥80% consensus).

ICF code	ICF category title	Round 1 (%)	Round 2 (%)	Round 3 (%)
**s410**	**Structure of the cardiovascular system**	**93**	**92**	**89**
**s430**	**Structure of the respiratory system**	**88**	**86**	**87**
**s750**	**Structure of the lower extremity**	**85**	**86**	**83**
**s760**	**Structure of the trunk**	79	77	**80**
s140	Structure of the sympathetic nervous system	75	73	78
s150	Structure of the parasympathetic nervous system	72	73	76
s730	Structure of the upper extremities	75	67	76
s740	Structure of the pelvic region	61	65	67
s110	Structure of the brain	66	61	65
s720	Structure of the shoulder region	57	55	63
s610	Structure of the urinary system	69	71	59
s710	Structure of the head and neck region	60	61	57
s120	Spinal cord and related structures	46	51	52

**Table 5:** International Classification of Functioning, Disability, and Health (ICF) activity and participation categories that were considered relevant by ≥50% of participants in the third round (items in bold with ≥80% consensus).

ICF code	ICF category title	Round 1 (%)	Round 2 (%)	Round 3 (%)
**d450**	**Walking**	**99**	**94**	**100**
**d530**	**Toileting**	**96**	**90**	**96**
**d550**	**Eating**	**97**	**94**	**96**
**d560**	**Drinking**	**97**	**94**	**94**
**d710**	**Basic interpersonal interactions**	**90**	**90**	**94**
**d230**	**Carrying out daily routine**	**91**	**92**	**91**
**d420**	**Transferring oneself**	**94**	**86**	**91**
**d570**	**Looking after one's health**	**93**	**88**	**91**
**d175**	**Solving problems**	**84**	**80**	**89**
**d177**	**Making decisions**	**85**	**88**	**89**
**d310**	**Communicating and receiving spoken messages**	**88**	**86**	**89**
**d540**	**Dressing**	**93**	**86**	**89**
**d330**	**Speaking**	76	78	**87**
**d620**	**Acquisition of goods and services**	76	76	**87**
**d760**	**Family relationships**	**88**	**90**	**87**
**d510**	**Washing oneself**	**88**	**84**	**85**
**d210**	**Undertaking a single task**	79	73	**83**
**d470**	**Using transportation**	**82**	78	**80**
**d520**	**Caring for body parts**	**84**	**80**	**80**
**d920**	**Recreation and leisure**	**82**	78	**80**
d115	Listening	78	78	78
d350	Conversation	**81**	78	78
d430	Lifting and carrying objects	70	63	78
d240	Handling stress and other psychological demands	**81**	**80**	76
d630	Preparing meals	**81**	77	76
d640	Doing housework	**81**	**80**	76
d740	Formal relationships	66	65	76
d110	Watching	69	65	74
d440	Fine hand use	64	61	74
d910	Community life	52	59	67
d860	Basic economic transactions	69	61	65
d140	Learning to read	55	57	63
d315	Communicating and receiving nonverbal messages	55	61	63
d335	Producing nonverbal messages	52	49	63
d720	Complex interpersonal interactions	54	51	63
d770	Intimate relationships	64	61	63
d220	Undertaking multiple tasks	63	55	61
d750	Informal social relationships	60	51	61
d870	Economic self-sufficiency	61	57	61
d155	Acquiring skills	58	49	59
d475	Driving	64	53	59
d850	Remunerative employment	51	49	57
d660	Assisting others	58	51	50
d940	Human rights	46	43	50

**Table 6:** International Classification of Functioning, Disability, and Health (ICF) environmental factor categories that were considered relevant by ≥50% of participants in the third round (items in bold with ≥80% consensus).

ICF code	ICF category title	Round 1 (%)	Round 2 (%)	Round 3 (%)
**e310**	**Immediate family**	**93**	**94**	**93**
**e410**	**Individual attitudes of immediate family members**	**93**	**84**	**93**
**e580**	**Health services, systems, and policies**	**85**	**86**	**85**
**e315**	**Extended family**	**84**	71	**84**
**e355**	**Health professionals**	**82**	78	**82**
**e575**	**General social support services, systems, and policies**	**82**	**84**	**82**
**e570**	**Social security services, systems, and policies**	**81**	**80**	**81**
e325	Acquaintances, peers, colleagues, neighbours, and community members	79	73	79
e320	Friends	78	71	78
e340	Personal care providers and personal assistants	76	**80**	76
e540	Transportation services, systems, and policies	72	73	72
e115	Products and technology for personal use in daily living	70	77	70
e120	Products and technology for personal indoor and outdoor mobility and transportation	70	77	70
e450	Individual attitudes of health professionals	63	65	63
e525	Housing services, systems, and policies	63	73	63
e440	Individual attitudes of personal care providers and personal assistants	61	67	61
e125	Products and technology for communication	60	61	60
e535	Communication services, systems, and policies	58	63	58
e110	Products or substances for personal consumption	57	57	57
e420	Individual attitudes of friends	54	53	54
e455	Individual attitudes of other professionals	54	53	54
e330	People in positions of authority	52	57	52

## Data Availability

The data used to support the findings of this study are available from the corresponding author upon request.
